# Evolution towards Linguistic Coherence in Naming Game with Migrating Agents

**DOI:** 10.3390/e23030299

**Published:** 2021-02-28

**Authors:** Dorota Lipowska, Adam Lipowski

**Affiliations:** 1 Faculty of Modern Languages and Literature, Adam Mickiewicz University in Poznań, 61-874 Poznań, Poland; 2 Faculty of Physics, Adam Mickiewicz University in Poznań, 61-614 Poznań, Poland; lipowski@amu.edu.pl

**Keywords:** multiagent modeling, migration, naming game, language formation

## Abstract

As an integral part of our culture and way of life, language is intricately related to the migrations of people. To understand whether and how migration shapes language formation processes, we examine the dynamics of the naming game with migrating agents. (i) When all agents may migrate, the dynamics generates effective surface tension that drives the coarsening. Such behaviour is very robust and appears for a wide range of densities of agents and their migration rates. (ii) However, when only multilingual agents are allowed to migrate, monolingual islands are typically formed. In such a case, when the migration rate is sufficiently large, the majority of agents acquire a common language that spontaneously emerges with no indication of surface-tension-driven coarsening. Relatively slow coarsening that takes place in a dense static population is very fragile, and an arbitrarily small migration rate can most likely divert the system towards the quick formation of monolingual islands. Our work shows that migration influences language formation processes, but additional details such as density or mobility of agents are needed to more precisely specify this influence.

Although there is a multitude of factors that shape our language, including culture, politics, economy, geography, or technology, the most important is the mutual interactions between multiple language users. It is, thus, tempting to examine language formation and its evolution using multiagent models and statistical mechanics methodology [1,2,3,4]. Valuable insight into language emergence [5], death [6], its diversification [7], importance [8], or appearance of grammar or linguistic categories [9] proves that such an approach is indeed promising.

An important factor that affects various aspects of our life is migration. This process may mix or separate human communities; thus, language formation processes are strongly influenced by it [10,11]. Moreover, some modern trends related mainly with globalisation most likely increase people’s migrations [12]. It is desirable to examine models that consider both linguistic interactions and migration, and thus gain some understanding of how related these two processes are. A possible candidate is a suitably extended naming game that proved to be useful in the studies of various aspects of the emergence of linguistic coherence [13,14]. A question is how coarsening, which in the naming game is basically similar to that in the Ising model, is affected by migration. Such similarity is related to the fact that the dynamics of both models are driven by surface tension [15], even though, in the naming game, it is rather effective [16]. Recent studies indicate that the dynamics of English dialect evolution is also driven by certain effective surface tension [17,18]. It is desirable to check to what extent the surface-tension-driven dynamics of the naming game is robust with respect to agent migration.

An interesting multiagent model where migration plays an important role was proposed some time ago by Schelling [19]. In his model, an agent was relocated if the number of its neighbours with the same orientation (an opinion or race) as the agent is below a certain threshold. Numerous versions of the Schelling model showed that the phase separation (formation of a ghetto) appears to be a very robust feature of the dynamics [20]. In the Schelling model, only those agents for which certain conditions are satisfied may migrate. Linguistic factors such as acquiring a new language might also influence our ability or willingness to relocate.

In the present paper, we examine the naming game with migration and address some of the above issues. In particular, we examine whether effective surface tension persists in such systems. We also examine the implications of state-dependent migration, but its applicability to real social systems is limited. Our simulations show that state-dependent diffusion usually leads to spatial segregation, but when sufficiently strong, it can trigger the formation of the dominant language in the entire system.

## 1. Model

In our model, we consider a population of agents placed on a square lattice of linear size *N* (with periodic boundary conditions). Each agent has its own inventory that is a dynamically modified list of words. Initially, agents are uniformly distributed on the lattice with density or probability ρ (double occupancy excluded). The dynamics of our model combines the lattice gas random migration with the so-called minimal version of the naming game [21,22].

More specifically, in an elementary step, an agent and one of its (four) neighbouring sites are randomly selected. With probability *d*, the agent migrates to the selected neighbouring site, provided that the chosen site is empty, and some additional conditions (dependent on the number of words in an agent’s inventory) are met. With probability 1−d, and provided that the selected neighbouring site is occupied, the chosen agent becomes the Speaker, its neighbour becomes the Hearer, and they play the naming game:

- The Speaker randomly selects a word from its inventory (or invents a new word if its inventory is empty) and transmits it to the Hearer. To invent a word, all agents have *M* different words at their disposal, and one of them is randomly selected.
- If the Hearer has the transmitted word in its inventory, the interaction is a success, and both players maintain only the transmitted word in their inventories.
- If the Hearer does not have the transmitted word in its inventory, the interaction is a failure, and the Hearer updates its inventory by adding this word to it.

The unit of time (t=1) is defined as $\rho N^{2}$ elementary steps that correspond to a single (on average) update of each agent. Agents may have in their inventories at most M≥2 different words, but the coarsening dynamics of models with M=2 and M>2 is similar to some extent. Indeed, the evolution towards one of its *M* absorbing states is driven by effective surface tension [1,23], and analogous similarities are between the Ising and Potts models [24]. In the following, we also refer to words communicated by agents as languages.

Recently, we analysed a naming-game model with migration in which relocation depended on the language used by an agent [22]. In this model, all agents were allowed to migrate (albeit with a language-dependent rate), and the main objective of this study is to demonstrate a certain symmetry breaking induced by difference in mobility. In the present paper, state-dependent mobility depends on the number of languages known by an agent, and not on the particular language used by the agent. Some other systems with migrating agents, but with different ordering dynamics (the voter model), were also analysed [25]. The emergence of consensus in the population was also examined in the case of agents moving in a continuous space [26] and in some robotic swarms [27]. It would be interesting to replace the local migration of our agents with the possibility of longer-distance steps, such as in some dynamical models defined on spatial networks [28].

Migration often refers to the mass movement of people in a certain direction or location, e.g., in China during the Qing dynasty [29]. To model such phenomena, a random walk of our agents has to be considerably modified.

## 2. Results

The naming game typically evolves towards a linguistic-consensus state where all agents have only a single word in their repositories, and thus every communication attempt results in success. Before reaching such a state, monolingual domains are formed, and their coarsening, driven by effective surface tension, eventually leads to the emergence of a linguistic consensus. The surface tension is known to drive the dynamics of many other models, e.g., the Ising or Potts model [15], and its absence, e.g., in the voter model [30], results in much different dynamics. In surface-tension-driven dynamics, correlation length can be related to the total length of domain boundaries, which can be easily extracted from the model configuration. In the naming game, competition between languages that takes place at domain boundaries implies that such interfacial agents are typically bi-(or more)lingual. Their concentration can be easily measured numerically and, being related to the total length of domain boundaries, it determines the correlation length in the system.

### 2.1. State-Independent Migration

**Figure 1:**
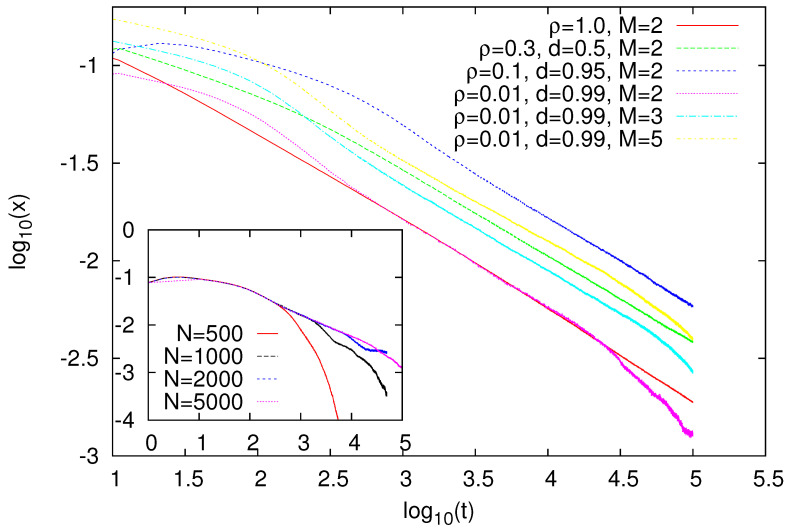
Time dependence of fraction *x* of bilingual agents for each set of parameters averaged over 100 independent runs. For ρ≥0.1, results were obtained for system size N=2000, and we checked that it was sufficiently large to avoid noticeable finite-size effects at the examined time scale. For ρ=0.01, results are shown for N=5000, and some late-time bending is still noticeable. Finite-size effects for ρ=0.01, d=0.99, and M=2 are illustrated in the inset. N≥1000 shows a transient slowdown in the decay of *x*, which we attribute to the formation of stripelike structures [32]. For increasing *N*, the influence of such stripes on fraction *x* seemed to diminish, and power-law decay set in.

The case of our main concern is such that only agents with two or more words in their inventories can migrate. However, first we report some results for the case where all agents are able to migrate. We performed simulations for several values of ρ and *d*, and measured fraction *x* of agents with two or more words in their inventories. The results of our simulations are presented in Figure 1. Most of our simulations were performed for M=2, but some results for M=3 and M=5 showed similar behaviour. For ρ=1, when the lattice was fully occupied by agents, and thus they had no space to migrate, *x* showed a power-law decay $x∼t^{-\alpha }$. From our data, we estimated α≈0.45(2), which agreed with some previous studies on the naming game [31,32] or related models of opinion formation [23,33]. For ρ<1, and in the presence of migration (d>0), fraction *x* of multilingual agents seemed to exhibit nearly the same asymptotic decay. This was even in the case when the lattice was sparsely covered with agents (ρ=0.01) that were engaged much more often in migration than they were in the naming game (d=0.99). In the Ising model with nonconservative dynamics, the length of the interface was related to excess energy above ground-state energy, and it decays as $t^{-1/2}$ (that easily translates into a $∼t^{1/2}$ increase in correlation length). The decay of *x* observed in the naming game is very similar and is most likely related to a certain effective surface tension generated in this kind of model [16].

Our simulations showed that surface-tension-driven dynamics in the naming game is very robust with respect to the concentration of agents and migration rate. There are some reasons to believe that some other factors do not change the qualitative features of the dynamics of such systems either. Indeed, we recently showed that surface-tension-driven dynamics is restored in the voter model (which has very different dynamics and no surface tension [30]) with only a small fraction of sites evolving according to Ising heat-bath dynamics [34]. It is, thus, plausible that, even in heterogeneous systems where some of our naming-game agents would be replaced with agents with dynamics similar to those in the voter model, the system would still exhibit surface-tension characteristics. Provided that the naming game mimics real linguistic interactions to some extent, such strong robustness suggests that surface-tension-driven dynamics could operate in (real) processes responsible for the evolution of natural languages, which certainly complies with recent analysis indicating that effective surface tension shapes patterns of dialect changes [17,18].

### 2.2. State-Dependent Migration

Agents with two or more words in their inventories, of which the fraction is denoted by *x*, are typically located at the interface of monolingual domains, and thus may be considered as multilingual. Since the ability (or willingness, or need) to migrate is not necessarily homogeneous in the population, we examine the case when only such multilingual agents may migrate.

Such state-dependent migration resembles the Schelling model of ghetto formation where an agent is relocated if the number of its neighbours of the same (as the agent) orientation is too small [19,20]. In the Schelling model, the orientation of an agent is fixed during the evolution of the model, which is not the case in our model. In such an analogy, our model could be considered as driven by nonconservative dynamics. Simulations of such a model reveal that both density ρ and migration rate *d* influence the dynamics of the model and its final state. We performed simulations in the low- (ρ=0.3) and high-density (ρ=0.8) regimes. In these regimes, there are some qualitative differences in the behaviour of the model that we describe below.

#### 2.2.1. ρ=0.3

**Figure 2:**
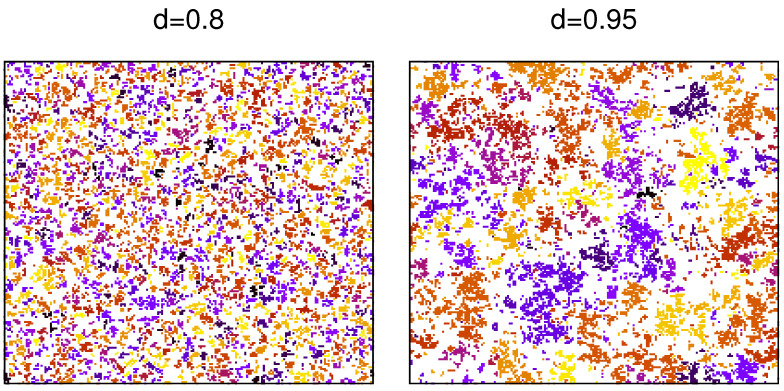
Spatial distribution of agents and languages they use after $t=10^{3}$ for d=0.8, and after $t=10^{4}$ for d=0.95. After such transients, all agents form separated clusters, become monolingual, and thus immobile (see Figure 4). Simulations were performed for N=200 and ρ=0.3; different colours correspond to different languages.

In this subsection, we describe our results in a low-density regime, and as a representative value, we chose ρ=0.3 and M=1000. Simulations showed that, in this case, after a relatively short transient, agents rearranged and formed monolingual clusters separated from each other (Figure 2).

**Figure 3:**
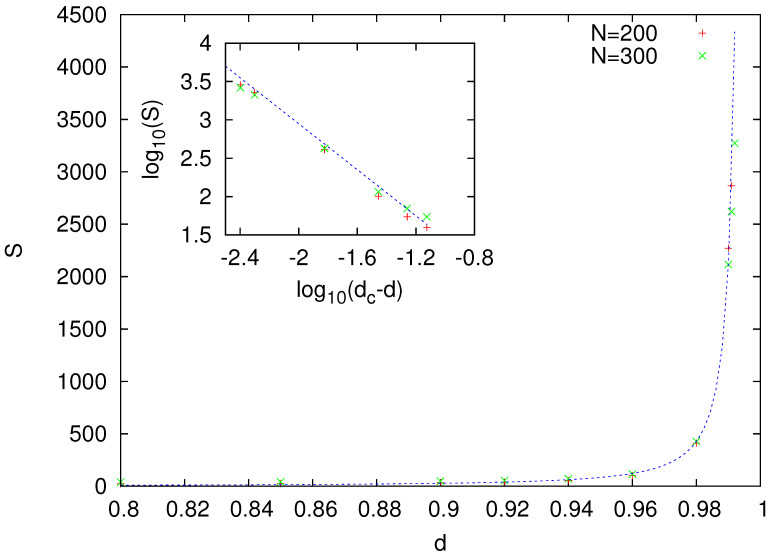
Average size of surviving languages as function of mobility *d*. (inset) Same data on a logarithmic scale. Least-squares fit shows that numerical data follow power-law divergence $S∼{\left(d_{c}-d\right)}^{-\gamma }$ with $d=d_{c}=0.995\left(1\right)$ and γ=1.5(1) (dashed line).

The size of these clusters seemed to increase with migration rate *d*. Calculation of the average size of such clusters *S* suggests that *S* may diverge at value $d=d_{c}$, which is close to but smaller than d=1 (Figure 3).

**Figure 4:**
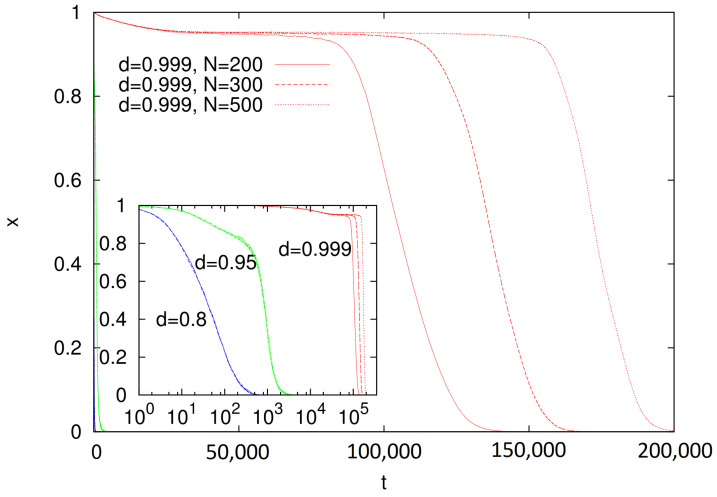
Time dependence of fraction *x* of mobile agents calculated for d=0.999 (red), 0.95 (green), and 0.8 (blue). Simulations performed for ρ=0.3 and system size N=200 (continuous lines), 300 (dashed), and 500 (dotted). Presented results are averages over 30 independent runs. For d=0.8 and 0.95, the decay of *x* took place on a short time scale and was nearly size-independent. (inset) Same data but with logarithmic time axis.

It is interesting to examine the evolution of our model for $d_{c}<d<1$. In particular, for d=0.999, simulations showed that the fraction of mobile agents *x* dropped to 0, but on a time scale that showed pronounced size dependence (Figure 4). For d=0.8 and 0.95, no such dependence was observed (inset in Figure 4).

**Figure 5:**
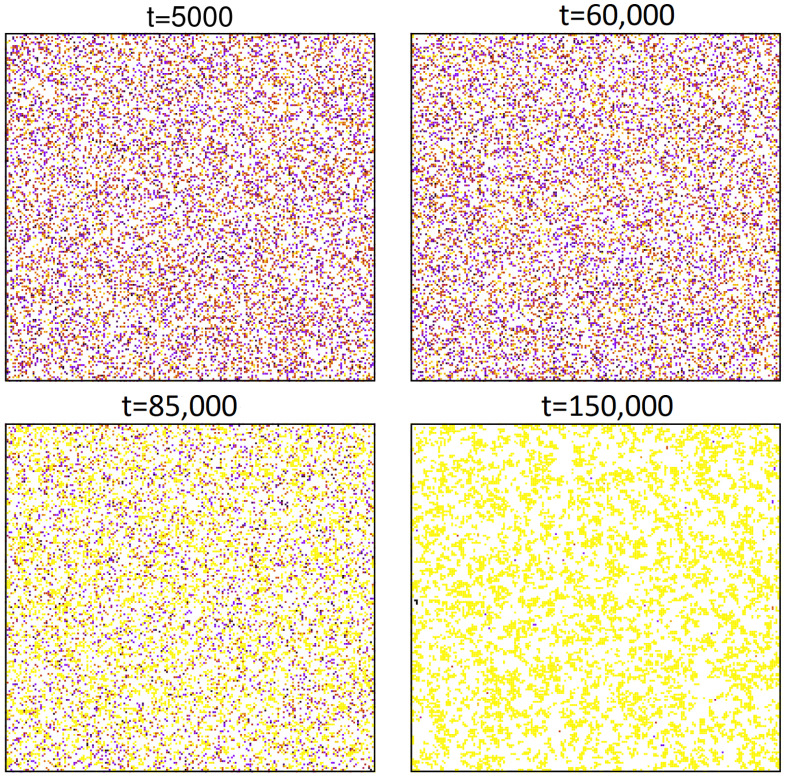
Snapshots of spatial distribution of agents and languages they use for d=0.999, ρ=0.3, and N=200. Dominant language emerges (around t=85000) without any indication of Ising-like coarsening.

According to Figure 3, for d=0.999, the average size of a cluster is infinite, which indicates the formation of a dominant language that is used by the majority of agents. Visual analysis of the time evolution of the model for d=0.999 showed that, in this case, the dominant language emerged without any indication of coarsening, which is characteristic to the naming game and other surface-tension-driven models (Figure 5). This suggests that a sufficiently strong state-dependent diffusion diminishes surface tension (to zero?), and the dominant cluster emerges in the system similarly as it does in the (multistate) voter model. The time scale of such a transition, possibly diverging with system size (Figure 4), supports such an interpretation. However, estimated critical value $d_{c}$ is very close to 1. Although the size of the surviving languages (Figure 3) for both N=200 and 300 seems to diverge at nearly the same value $d=d_{c}=0.995\left(1\right)$, we cannot exclude that this is actually the finite-size effect and, in the thermodynamic limit, $d_{c}=1$. However, the true thermodynamic limit is perhaps not that important in the linguistic context, and the dynamics that we observed might also be relevant and interesting as a finite-size effect.

#### 2.2.2. ρ=0.8

**Figure 6:**
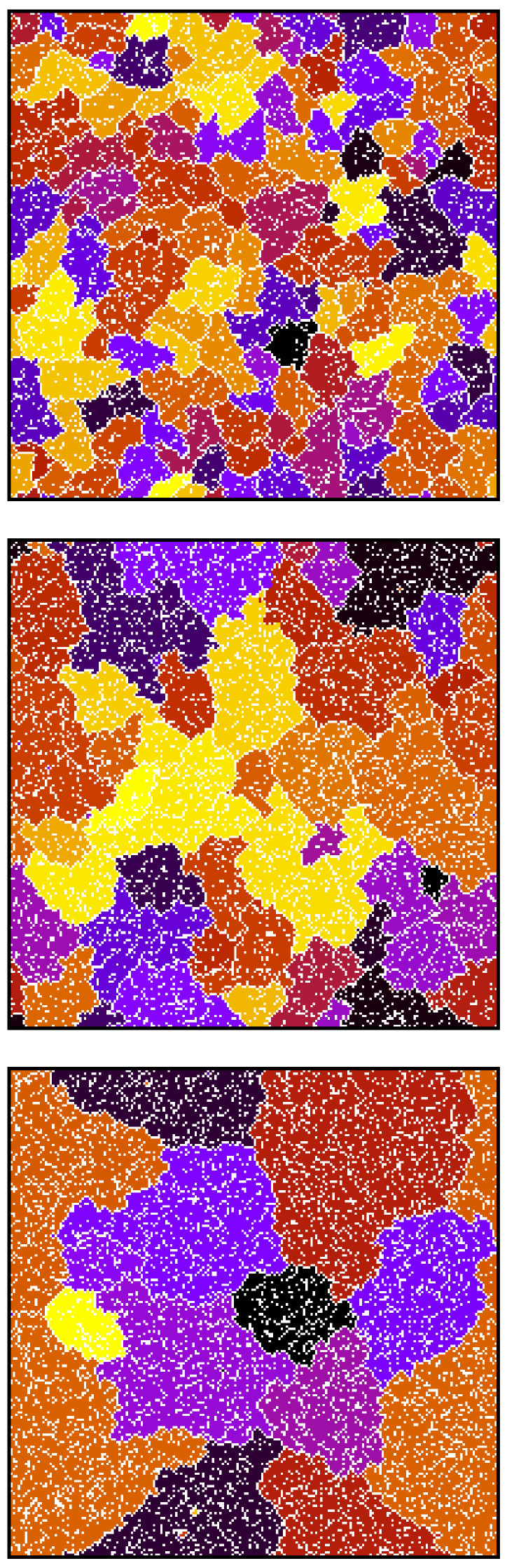
Final spatial distributions of agents and languages they use for ρ=0.8, N=200, M=1000, and d=0.1 (**top**), d=0.01 (**middle**), and d=0.001 (**bottom**).

In this subsection, we examine the behaviour of our model for larger density, namely, for ρ=0.8. Similarly to the ρ=0.3 case, migration coupled with the ordering mechanism of the naming game lastly led to the formation of monolingual islands separated by some empty spaces; examples of such structures are shown in Figure 6. The decrease in migration rate *d* resulted in an increase in island size. This is contrary to the ρ=0.3 case, where the size of islands was increasing with the increase in *d*. However, configurations presented in Figure 6 were obtained for small values of migration rate *d*. For larger *d* (close to d=1), a trend similar to that for ρ=0.3 would perhaps be observed. Since the system with ρ=0.8 was relatively dense, the numerical investigation of such a regime might be difficult.

**Figure 7:**
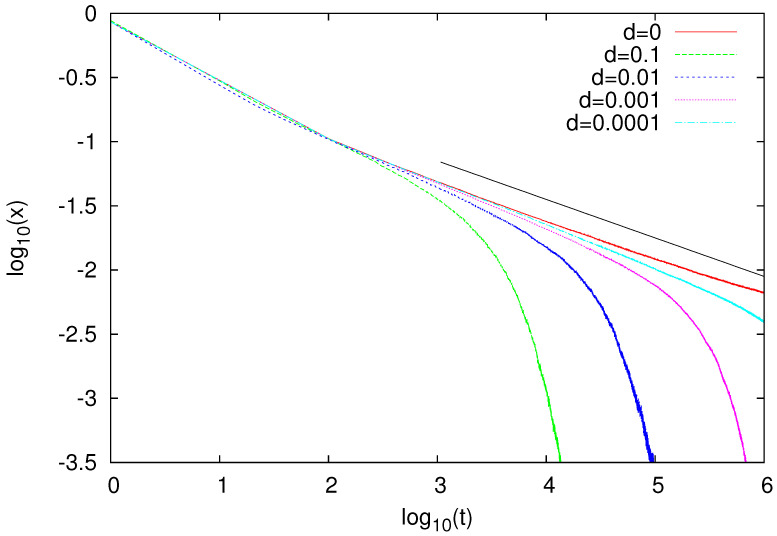
Time dependence of fraction *x* of bilingual agents calculated for ρ=0.8, N=1000, and several values of mobility *d*. Solid straight line has slope corresponding to $x∼t^{-0.3}$, and some bending of our data indicates that asymptotic decay for d=0 might be even slower than that. Presented results were averaged over 100 independent runs.

For N=1000 and M=1000, we also calculated the time dependence of *x* (Figure 7). In the absence of migration (d=0), we observed a power-law-like decay of *x*, but some bending of our data suggested that estimated decay $∼t^{-0.3}$ may not be truly asymptotic. Slower decay, perhaps logarithmically slow, would actually be consistent with the coarsening of the Ising model on diluted lattices [35]. Leaving aside the correct asymptotic form of the decay of *x*, the d=0 coarsening that took place in our model for ρ=0.8 was an expected feature, assuming some analogy to the Ising model, with which the naming game seems to have some similarities. Indeed, above the site-percolation threshold ($\rho_{c}\approx 0.5928$ [36,37]), the Ising model was expected to ferromagnetically order at a sufficiently low temperature with the accompanying power-law coarsening [38,39], and we expect similar behaviour in the naming game. Below such a threshold ($\rho <\rho_{c}$), our agents were located in finite clusters, which rather quickly became monolingual due to the naming game.

For d>0, *x* showed rapid (probably faster than the power-law) decay (Figure 7). We associate such a decay with the formation of monolingual islands (Figure 6). What is surprising is that even a very small migration rate *d* is sufficient to bring the system to such a multi-island configuration. Indeed, even for $d=10^{-4}$, data seem to veer off the d=0 line. With *d* decreasing, this deviation takes place at an increasing time scale, which is related to the formation of islands of increasing size.

**Figure 8:**
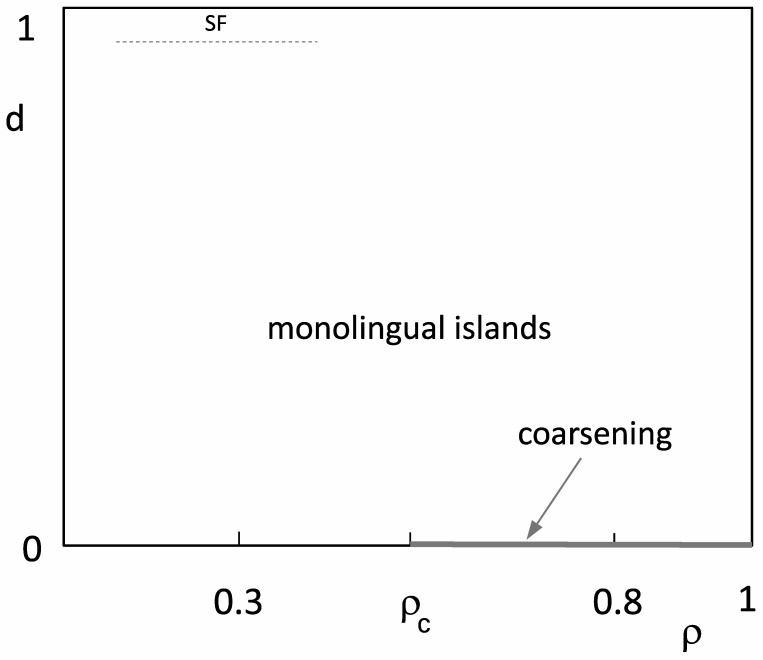
(ρ,d) phase diagram as inferred from our simulations. In the largest portion of the phase diagram, the final state was quickly reached, comprising finite-size monolingual islands. We expected power-law coarsening only for d=0 and $\rho >\rho_{c}$, and for ρ<1, the coarsening was probably slower than $t^{1/2}$ due to random dilution. For *d* very close to 1, we expected the regime where the dominant language is formed via spontaneous fluctuation (SF, dashed line is not in scale).

The overall behaviour of our model for state-dependent migration is presented in the phase diagram in Figure 8.

## 3. Conclusions

In summary, we examined how ordering dynamics of the naming game is affected by migration. In the version where all agents are allowed to migrate, the coarsening of our model indicated the presence of effective surface tension. Such behaviour is very robust with respect to the concentration of agents or their migration rate. Recently, we showed that effective surface tension appears in a heterogeneous voter model, with a small fraction of agents operating with Ising heat-bath dynamics [34]. Since the naming game shares some similarity with the Ising model, effective surface tension should be a generic feature of language-formation models that would be resilient against dilution, migration, or dynamical heterogeneities. This very much agrees with recent analysis where surface tension shaped English dialect evolution [17,18]. It was also suggested [18] that the diffusion of language users may reduce surface tension and shift the dynamics towards being voterlike. Our simulations did not support such behaviour, at least within the scope of our model.

When only multilingual agents were allowed to migrate, we observed the formation of monolingual islands. Such state-dependent migration dynamics resembles that of the Schelling model, and islands may be considered as analogues of ghettos that are typically formed in this model. Similarly to the Schelling model, the formation of islands is a robust feature of the dynamics, and it takes place for small and large concentrations of agents. Unlike the Schelling model, our agents might change their language; in this respect, they are driven by nonconservative dynamics. Our simulations suggested that, when the state-dependent migration rate is sufficiently large, a certain language becomes dominant and spreads over the majority of agents. However, transition towards such linguistic coherence is not surface-tension-driven coarsening, but rather spontaneous fluctuation, similar perhaps to the transition in the voter model. The predicted migration-induced reduction of surface tension [18] would thus take place, but only with the migration of multilingual agents. When the concentration of agents is above a site-percolation threshold, and state-dependent migration is absent, agents form an infinite cluster, and naming-game dynamics induces coarsening, albeit slower than that on an undiluted lattice. Such coarsening appears to be very fragile with respect to state-dependent migration, and arbitrarily small migration most likely directs the dynamics towards the formation of monolingual islands.

Migration is an important factor that should be considered in studying language-formation models and other agreement-dynamics systems. It is desirable to develop alternative approaches that allow for at least a qualitative understanding of our results, which are only based on numerical simulations. Field-theory techniques based on the Fokker–Planck equation were used to analyse a related class of models, the so-called voter model with intermediate states [33]; it would be interesting to develop a similar approach in the context of our models. However, taking into account the migration of our particles is likely to result in a more complex field-theory description. It would also be interesting to examine whether effective surface tension also appears in reinforcement-learning systems with migration [40] or in heterogeneous systems where agents evolve with different kinds of dynamics. The elucidation of the role of state-dependent migration in the formation of a dominant language, or in the high fragility of slow coarsening on a diluted lattice would also be desirable.

## Data Availability

Not applicable.
